# Optimal Nitrogen Application Strategies for Alfalfa Under Different Precipitation Patterns: Balancing Yield, Nitrogen Fertilizer Use Efficiency, and Soil Nitrogen Residue

**DOI:** 10.3390/plants15020333

**Published:** 2026-01-22

**Authors:** Yanbiao Wang, Yuanbo Jiang, Haiyan Li, Boda Li, Jinxi Chen, Minhua Yin, Yanxia Kang, Guangping Qi, Yanlin Ma, Bojie Xie, Haoxiang Jin, Tongjin Wu, Shan Li

**Affiliations:** College of Water Conservancy and Hydropower Engineering, Gansu Agricultural University, Lanzhou 730070, China; 1073323020378@st.gsau.edu.cn (Y.W.); 1073323010121@st.gsau.edu.cn (Y.J.); 107332201100@st.gsau.edu.cn (H.L.); 1073324020375@st.gsau.edu.cn (B.L.); 1073324120804@st.gsau.edu.cn (J.C.); qigp@gsau.edu.cn (G.Q.); mayl@gsau.edu.cn (Y.M.); jiebj@gsau.edu.cn (B.X.); 20222202046@st.gsau.edu.cn (H.J.); 20221520117@st.gsau.edu.cn (T.W.); 20232202067@st.gsau.edu.cn (S.L.)

**Keywords:** precipitation pattern, yield, nitrogen fertilizer regulation, NO_3_^−^–N, NH_4_^+^–N, APSIM model

## Abstract

Rational nitrogen applications can not only improve nutrient use efficiency, but also reduce environmental pollution caused by nitrogen leaching. To explore reasonable nitrogen application strategies for synergistically enhancing alfalfa production and ecological benefits, this study calibrated and validated the APSIM–Lucerne model based on field experiments conducted from 2021 to 2023. The effects of nitrogen application levels of 0, 80, 120, 140, 160, 180, 200, and 240 kg/ha on alfalfa yield, soil NO_3_^−^–N and NH_4_^+^–N residues, and nitrogen use efficiency under dry, normal, and wet years were simulated. The results indicate: (1) The calibrated APSIM–Lucerne model effectively simulates alfalfa yield and soil nitrogen residuals (*R*^2^ ranging from 0.67 to 0.91, NRMSE between 6.55% and 24.03%). (2) Increased nitrogen application significantly elevates soil nitrogen residue, yet alfalfa yield follows a pattern of initial increase followed by decline, with nitrogen fertilizer use efficiency continuously decreasing. Under identical nitrogen application rates, the wet year type proves more advantageous for achieving high yields, low nitrogen residue, and high nitrogen fertilizer use efficiency. (3) The nitrogen application thresholds for achieving increased alfalfa yields and high efficiency during dry years, normal years, and wet years are 107–140 kg/ha, 135–160 kg/ha, and 150–183 kg/ha, respectively.

## 1. Introduction

Fertilization is one of the most direct and effective measures to increase crop yield [1]. Since the early 1970s, the application of chemical fertilizers in China has consistently increased, playing a crucial role in boosting grain production and ensuring national food security [2,3]. However, excessive application of chemical fertilizers (especially nitrogen fertilizers) has resulted in large quantities of nitrogen not being absorbed by plants remaining in the soil in the form of nitrate nitrogen (NO_3_^−^–N) and ammonium nitrogen (NH_4_^+^–N) [4]. NO_3_^−^–N readily leaches with rainfall or irrigation water, not only reducing fertilizer efficiency but also causing degradation of arable land quality and triggering non-point source pollution problems such as groundwater nitrate contamination, lake cyanobacterial blooms, and marine red tides [5,6]. Excess NH_4_^+^–N in soil promotes the emission of nitrous oxide (N_2_O), a potent greenhouse gas, through nitrification and denitrification processes, thereby contributing to global warming [7,8]. Furthermore, excessive nitrogen fertilizer application substantially raises production costs and diminishes fertilizer use efficiency, conflicting with the modern agricultural development principles that prioritize “green, efficient, and sustainable” practices. In China’s agricultural production system, forage crops, particularly alfalfa and other quality fodder, occupy an important position, and nitrogen management in their production processes directly affects livestock development and the ecological health of agricultural fields [9]. Therefore, establishing precise nitrogen regulation strategies based on actual forage crop requirements and aligned with environmental conditions is crucial for simultaneously enhancing the economic benefits of alfalfa production and ensuring safety.

The core of optimizing nitrogen fertilizer management lies in deeply analyzing the interaction mechanisms of the complex “crop–soil–climate” system. Precipitation, as a key climatic factor, directly influences physical, chemical, and biological processes such as soil nitrogen mineralization, nitrification, denitrification, leaching, and crop nitrogen uptake and utilization by regulating soil water conditions [10,11]. Different precipitation patterns govern variations in soil water surplus and deficit, thereby shaping the distinct characteristics of nitrogen transformation processes. In dry years, limited soil water reduces nitrogen mineralization rates, with more nitrogen remaining in organic form; in wet years, sufficient water simultaneously accelerates mineralization and nitrification, yet is accompanied by substantial leaching and denitrification, leading to sudden losses of “nitrogen leaving with water”; normal years present a relatively balanced state between nitrogen mineralization and crop uptake with moderate soil nitrogen residue [12,13,14]. Under different combinations of precipitation patterns and nitrogen application rates, crop yield, soil nitrogen residue, and nitrogen fertilizer use efficiency show significant variations. Traditional field experiments suffer from low efficiency, substantial environmental disturbance, poor reproducibility, and limited spatial extrapolation capability [15]. Furthermore, constrained fertilizer application gradients and insufficient duration of fertilization treatments restrict their ability to systematically capture the effects of different precipitation patterns. Crop growth models are mathematical models that use mathematical concepts and information technology to dynamically and quantitatively simulate the physiological and ecological processes of crops from germination to maturity, as well as their final yield [16]. By integrating information on meteorology, soil, crop genetic characteristics, and agricultural management measures, and other aspects, these models predict crop growth under different environmental conditions and management strategies, thereby assisting farmland management and decision–making [17,18]. Crop growth models, when combined with field experiments, can effectively address the limitations that arise when relying solely on field experiments. The APSIM model is a dynamic mechanistic model jointly developed by the Australian Agricultural Production Systems Research Unit (APSRU) and the Australian Commonwealth Organization for Science and Industry (ACOSI) [18,19]. In contrast to mainstream models, such as WOFOST [20], DSSAT [21], and STICS [22], which primarily focus on simulating a single crop growth season, the APSIM model utilizes a highly modular structural design. This design allows APSIM to effectively simulate multi-crop rotation sequences and the dynamic processes of long-term agroecosystems. The model can comprehensively simulate key processes, including crop growth, soil water and nutrient transport, carbon and nitrogen cycling, and quantitatively assess the long-term impacts of different management practices (such as fertilization and irrigation) and climate change scenarios on agricultural systems [23]. Yin et al. [24] used the APSIM model in Dingxi, Gansu, to study optimal nitrogen application rates and depths that simultaneously increase wheat yield and nitrogen fertilizer use efficiency. Thorburn et al. [25] used the APSIM model in Australia to simulate sugarcane crop yield and nitrogen fertilizer use efficiency under different field management practices. Wu et al. [26] simulated spring wheat yield and nitrogen fertilizer use efficiency under different precipitation patterns in Inner Mongolia based on the APSIM model, optimizing spring wheat water–nitrogen management practices in that region. Currently, the APSIM model has been extensively applied and rigorously validated for simulating the growth and development processes of various annual grain, oilseed, and economic crops [27,28]. However, its application in perennial forages, particularly alfalfa, remains relatively limited, and systematic simulation studies focusing on alfalfa growth and nitrogen management across different precipitation patterns are notably scarce.

Alfalfa (*Medicago sativa* L.), recognized as the “king of forage crops” for being the earliest cultivated and most widely grown leguminous forage in the world [29], provides high–quality forage for ruminant animals due to its high protein content, good palatability, wide adaptability, and high yield [30]. Owing to its well-developed root system, alfalfa enhances soil fertility, increases organic matter content, and reduces the risk of topsoil degradation [31]. Gansu Province, leveraging its unique advantages in sunlight, heat, and land resources, has become China’s largest high–quality alfalfa production region, accounting for the country’s highest planting area with a proportion of approximately 60% [32]. However, under conditions of high-intensity harvesting utilization, poor soil conditions, and extensive nutrient management, alfalfa’s biological nitrogen-fixation capacity cannot meet the demand for sustained high yields, and its yield potential remains largely untapped. In light of this, this study aims to couple three years of field experiments with the APSIM–Lucerne model to: (1) calibrate and validate the applicability of the APSIM–Lucerne model in the research area using field experiment data; (2) simulate the effects of different precipitation patterns and nitrogen application rates on soil nitrogen residue, alfalfa yield, and nitrogen fertilizer use efficiency by integrating long–term historical meteorological data; (3) quantitatively analyze the response relationships of alfalfa yield, soil nitrogen residue, and nitrogen fertilizer use efficiency to nitrogen application rates, and propose nitrogen fertilizer optimization and regulation strategies for alfalfa under different precipitation patterns that balance high yield, high efficiency, and ecological safety, providing theoretical basis and technical support for alfalfa nitrogen management in Gansu’s Yellow River irrigation areas and similar ecological regions.

## 2. Results

### 2.1. Verification of the Applicability of the APSIM–Lucerne Model

#### 2.1.1. Yield Verification

**Figure 1:**
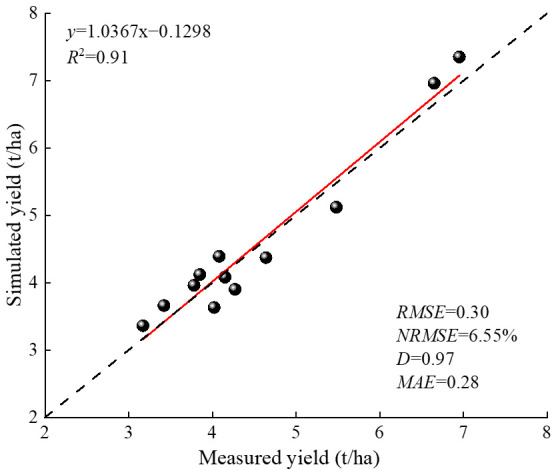
Linear regression fitting of the simulated values and measured values of alfalfa yield. In the figure, RMSE denotes root mean square error, NRMSE denotes normalized root mean square error, D denotes index of agreement, and MAE denotes mean absolute error. The dashed line represents the 1:1 reference line, the solid line represents the fitted line between simulated and observed values, and the black dots represent observed and simulated values.

Verification of the APSIM–Lucerne model based on 2023 field trial data showed high consistency between simulated and measured alfalfa yields. Correlation analysis (Figure 1) showed that both *R*^2^ and D values approached 1.0 (*R*^2^ = 0.91, D = 0.97), with NRMSE and MAE values of 6.55% and 0.28 t/ha, respectively, indicating that the model had high simulation accuracy for alfalfa yield, with small errors and good agreement between simulated and measured values.

#### 2.1.2. Validation of NO_3_^−^–N and NH_4_^+^–N Residual Content

**Figure 2:**
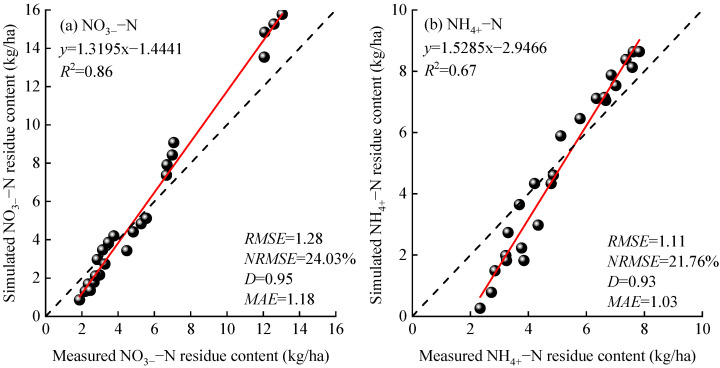
Linear regression fitting of the simulated and measured values of soil nitrogen residue. In the figure, RMSE denotes root mean square error, NRMSE denotes normalized root mean square error, D denotes index of agreement, and MAE denotes mean absolute error. The dashed line represents the 1:1 reference line, the solid line represents the fitted line between simulated and observed values, and the black dots represent observed and simulated values.

The linear fitting between the simulated and measured soil nitrogen residues in 2023 (Figure 2) shows that the *R*^2^ are 0.86 and 0.67, respectively, while the D values are 0.95 and 0.93. The NRMSE and MAE are 21.76%, 24.03%, and 1.18 kg/ha, 1.03 kg/ha, respectively. This indicates that the error between the simulated and measured soil NO_3_^−^–N residuals is small and the consistency is good, while the error for the soil NH_4_^+^–N residuals is larger, with only moderate consistency.

#### 2.1.3. Simulation of Alfalfa Yield Under Different Precipitation Patterns

**Figure 3:**
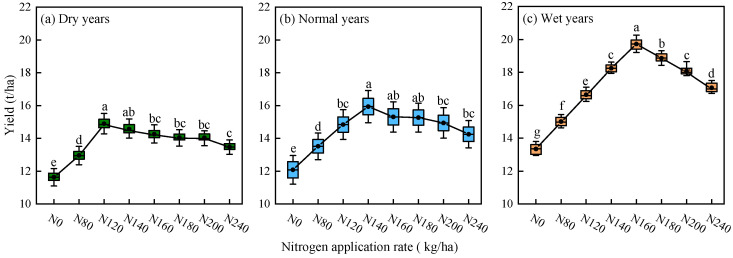
Alfalfa yield simulation under varied precipitation patterns and nitrogen application rates. Different lowercase letters indicate significant differences in alfalfa yield among different treatments (*p* < 0.05). Green indicates dry years, blue indicates normal years, and orange indicates wet years.

After calibrating the parameters of the APSIM–Lucerne model and evaluating its applicability, this study simulated alfalfa yields in the experimental area from 2000 to 2024 under different precipitation patterns and nitrogen application rates (Figure 3). Given that previous research has shown that alfalfa experiences a sharp decline in yield after continuous planting for 6–8 years [33], this study adopted a grouped simulation approach to avoid interference from this biological trait in long–term trend analysis. The simulation design divided the entire simulation period (2000–2024) into five consecutive 5-year periods. This effectively isolated the yield decline associated with plant aging, thereby enabling the model to more accurately elucidate the independent effects of different nitrogen application rates and precipitation patterns on alfalfa yield and nitrogen use efficiency.

From the figure, it can be observed that: (1) Alfalfa yield is significantly affected by both precipitation patterns and nitrogen input levels (*p* < 0.05). In wet years, alfalfa yields reach a maximum, are intermediate in normal years, and are lowest during dry years. Additionally, the yield responds to increasing nitrogen application in a non–linear fashion, initially increasing before subsequently declining. (2) In dry years, normal years, and wet years, alfalfa yield reaches its maximum under N120, N140, and N160 treatments, respectively, at 14.90, 16.19, and 19.72 t/ha, which is an increase of 2.83–28.00%, 5.10–31.29%, and 6.42–47.78% compared to other treatments, respectively.

#### 2.1.4. Simulation of Soil NO_3_^−^–N and NH_4_^+^–N Residuals Under Different Precipitation Patterns

**Figure 4:**
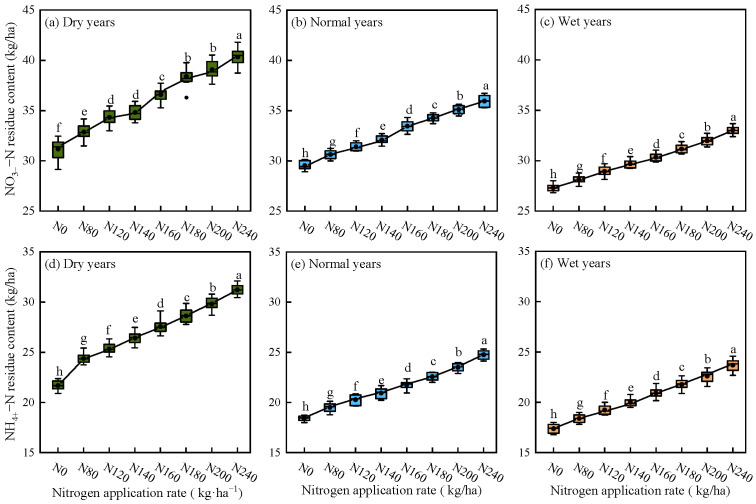
Simulation of soil nitrogen residues under different precipitation patterns and nitrogen application rates. Different lowercase letters indicate significant differences in soil nitrogen residue levels among treatments (*p* < 0.05). Green indicates dry years, blue indicates normal years, and orange indicates wet years.

As shown in Figure 4, the APSIM–Lucerne model simulated that the residual amounts of soil NO_3_^−^–N and NH_4_^+^–N in alfalfa fields significantly increase with the amount of nitrogen applied, exhibiting a pattern of wet years < normal years < dry years. With increasing nitrogen application, the residual amounts of NO_3_^−^–N and NH_4_^+^–N in dry years are much greater than those in normal and wet years. In dry years, normal years, and wet years, compared to the non-fertilized treatment, the soil NO_3_^−^–N and NH_4_^+^–N residuals increase by 29.40%, 21.77%, 20.90%, 44.05%, 34.42%, and 36.35%, respectively, when the nitrogen application rate is 240 kg/ha.

### 2.2. Alfalfa Nitrogen Fertilizer Use Efficiency Under Different Precipitation Patterns

**Figure 5:**
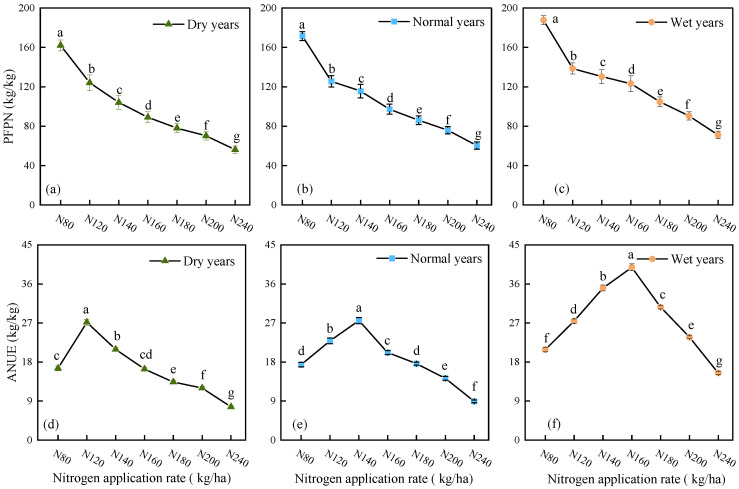
Nitrogen fertilizer use efficiency of alfalfa under different precipitation patterns and nitrogen application rates. In the figure, (**a**–**c**) represent PFPN for dry years, normal years, and wet years, respectively, while (**d**–**f**) represent ANUE for dry years, normal years, and wet years, respectively. Different lowercase letters indicate significant differences in alfalfa PFPN and ANUE among different treatments (*p* < 0.05).

At the same nitrogen application rate, the PFPN and ANUE of alfalfa exhibit a significant declining trend across the precipitation year-type gradient, with the highest values occurring in wet years, intermediate values in normal years, and the lowest values in dry years (Figure 5). Under the same precipitation year-type conditions, alfalfa’s PFPN shows a declining trend with increasing nitrogen application levels, while ANUE exhibits a single-peak pattern of increase followed by a decrease. PFPN under each year-type reaches its maximum at the N80 treatment, with improvements compared to other treatments of 30.53–188.53%, 36.54–184.45%, and 35.35–164.03% in dry years, normal years, and wet years, respectively. The optimal treatment for ANUE varies by year type: in dry years, the N120 treatment is highest, with an increase of 29.78–254.12% compared to other treatments; in normal years and wet years, both reach peak values at the N140 treatment, with increases of 20.42–208.59% and 13.60–157.33%, respectively.

### 2.3. Correlation Between Soil NO_3_^−^–N and NH_4_^+^–N Residuals and Nitrogen Fertilizer Use Efficiency

**Figure 6:**
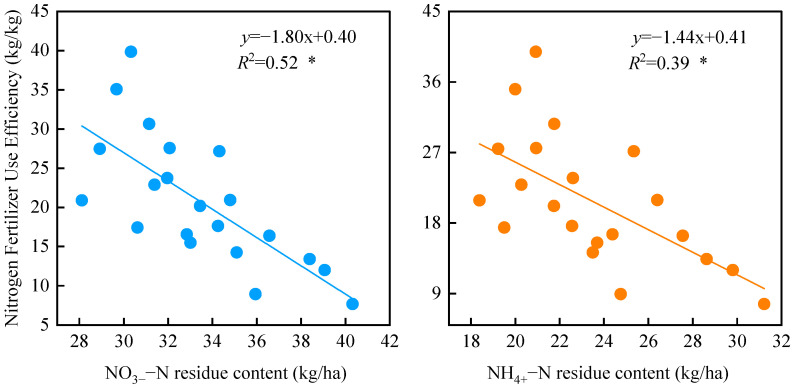
Correlation between nitrogen fertilizer use efficiency and soil nitrogen residue. In the figure, Blue indicates the correlation between NO_3_^−^–N residue levels and nitrogen fertilizer use efficiency, orange indicates the correlation between NH_4_^+^–N residue levels and nitrogen fertilizer use efficiency, and the straight line represents the correlation fit line. * indicates significant differences between different indicators (*p* < 0.05).

Correlation analysis (Figure 6) indicates a significant negative correlation between nitrogen fertilizer use efficiency (NUE) and both soil NO_3_^−^–N and NH_4_^+^–N residuals (*p* < 0.05). Notably, the correlation between NUE and NO_3_^−^–N residuals is stronger (*R*^2^ = 0.52) than that between NUE and NH_4_^+^–N residuals (*R*^2^ = 0.39).

### 2.4. Interrelationship Among Alfalfa Yield, Soil NO_3_^−^–N and NH_4_^+^–N Residuals, Nitrogen Fertilizer Use Efficiency, and Nitrogen Application Rate

**Figure 7:**
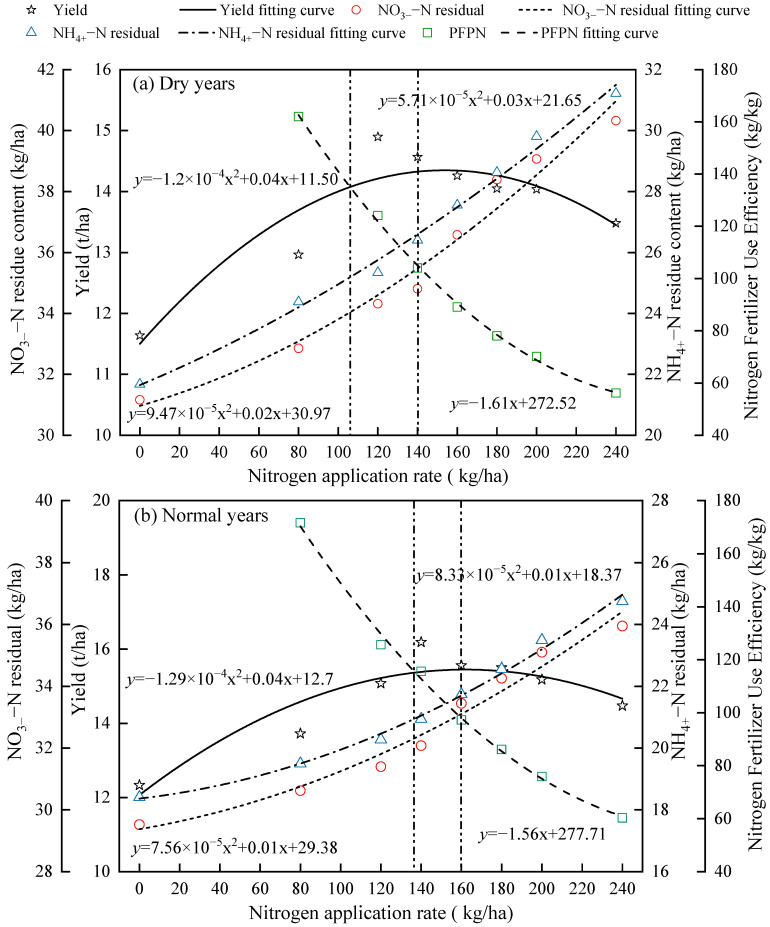

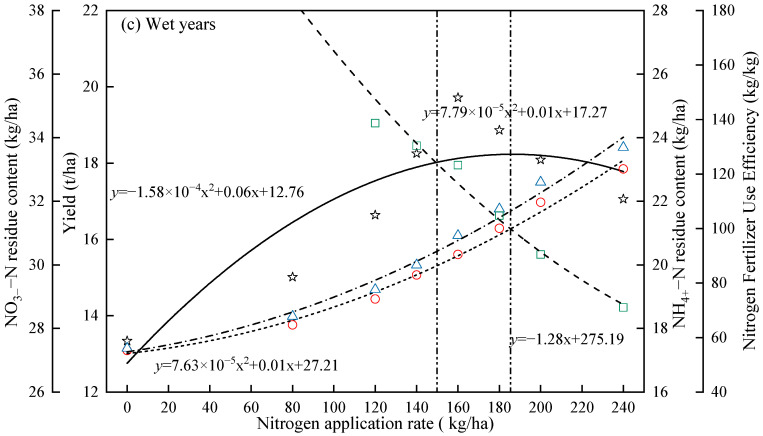
Interrelationship between alfalfa yield, soil nitrogen residues, nitrogen fertilizer use efficiency, and nitrogen application rates.

The simulations conducted with the APSIM–Lucerne model show significant correlations among alfalfa yield, soil NO_3_^−^–N and NH_4_^+^–N residuals, nitrogen fertilizer use efficiency, and nitrogen application rates (Figure 7). In dry years, alfalfa yield demonstrates a quadratic relationship with increasing nitrogen application. The fitted equation is given by y = −1.2 × 10^−4^x^2^ + 0.04x + 11.50 (*R*^2^ = 0.78, *p* < 0.05). When the nitrogen application exceeds 120 kg/ha, alfalfa yield does not significantly increase. The regression equation for soil NO_3_^−^–N residuals in relation to nitrogen application is y = 9.47 × 10^−5^x^2^ + 0.02x + 30.97 (*R*^2^ = 0.98, *p* < 0.001). The regression equation for soil NH_4_^+^–N residuals in relation to nitrogen application is y = 5.71 × 10^−5^x^2^ + 0.03x + 21.65 (*R*^2^ = 0.98, *p* < 0.001). Nitrogen fertilizer use efficiency decreases linearly with increasing nitrogen application, with the fitting equation given by y = −1.61x + 272.52 (*R*^2^ = 0.91, *p* < 0.05). The intersection points for yield, NO_3_^−^–N residuals, NH_4_^+^–N residuals, and nitrogen fertilizer use efficiency occur between nitrogen application rates of 107 kg/ha and 140 kg/ha, indicating a reasonable nitrogen application range for dry years.

In normal years, the relationship between alfalfa yield and nitrogen application is also quadratic. The fitted equation is expressed as y = −1.29 × 10^−4^x^2^ + 0.04x + 11.70 (*R*^2^ = 0.85, *p* < 0.05). When the nitrogen application exceeds 140 kg/ha, alfalfa yield does not significantly increase. The regression equation for soil NO_3_^−^–N residuals is y = 7.56 × 10^−5^x^2^ + 0.01x + 29.38 (*R*^2^ = 0.96, *p* < 0.001). The regression equation for soil NH_4_^+^–N residuals is y = 8.33 × 10^−5^x^2^ + 0.01x + 18.37 (*R*^2^ = 0.98, *p* < 0.001). Nitrogen fertilizer use efficiency decreases linearly with increasing nitrogen application, with the fitting equation given by y = −1.56x + 277.71 (*R*^2^ = 0.92, *p* < 0.05). The intersection points for yield, NO_3_^−^–N residuals, NH_4_^+^–N residuals, and nitrogen fertilizer use efficiency occur between nitrogen application rates of 135 kg/ha and 160 kg/ha, indicating the reasonable nitrogen application range for normal years.

In wet years, the relationship between alfalfa yield and nitrogen application is again quadratic, with the fitting equation given by y = −1.58 × 10^−4^x^2^ + 0.06x + 12.76 (*R*^2^ = 0.78, *p* < 0.05). When the nitrogen application exceeds 160 kg/ha, alfalfa yield does not significantly increase. The regression equation for soil NO_3_^−^–N residuals is y = 7.63 × 10^−5^x^2^ + 0.01x + 27.21 (*R*^2^ = 0.98, *p* < 0.001). The regression equation for soil NH_4_^+^–N residuals is y = 7.79 × 10^−5^x^2^ + 0.01x + 17.27 (*R*^2^ = 0.98, *p* < 0.001). Nitrogen fertilizer use efficiency decreases linearly with increasing nitrogen application, with the fitting equation given by y = −1.28x + 275.19 (*R*^2^ = 0.98, *p* < 0.001). The intersection points for yield, NO_3_^−^–N residuals, NH_4_^+^–N residuals, and nitrogen fertilizer use efficiency occur between nitrogen application rates of 150 kg/ha and 183 kg/ha, indicating a reasonable nitrogen application range for wet years.

## 3. Discussion

### 3.1. Evaluation of Model Adaptability

The APSIM model is a process-driven, highly modular, and open-source crop growth simulation tool that demonstrates significant advantages in crop production simulation (predicting crop yield, biomass, phenological stages, etc.), soil process simulation (water balance, carbon and nitrogen cycling), and management measure assessment. Based on meteorological, soil, and field management data collected from 2021 to 2023, this study used observations from 2021 to 2022 for APSIM–Lucerne model parameter calibration and reserved the 2023 data for independent validation. The results indicated that the *R*^2^ and NRMSE for alfalfa yield simulation were 0.91 and 6.55%, respectively, which are similar to the results obtained by Yang et al. [34] and Zhu et al. [35] for alfalfa (*R*^2^ = 0.81~0.98, NRMSE = 10.57%~22.48%), Chimonyo et al. [36] for sorghum–cowpea intercropping (*R*^2^ = 0.86~0.98), and Shukr et al. [37] for cotton (*R*^2^ = 0.82~0.93). Additionally, the *R*^2^ and NRMSE for simulating NO_3_^−^–N and NH_4_^+^–N residuals in this study were 0.86, 0.67, 21.76%, and 24.03%, respectively. These results are consistent with those of Jin et al. [1], who utilized the APSIM model to simulate soil NO_3_^−^–N residuals in dryland corn fields in Shouyang, Shanxi (*R*^2^ = 0.85, NRMSE = 15%). They also align with the findings of Tahir et al. [38], who employed the APSIM model to predict NO_3_^−^–N and NH_4_^+^–N concentrations in black soil in Yangling, Jilin, achieving *R*^2^ values close to 1. This indicates that the APSIM–Lucerne model can accurately simulate alfalfa yield and the accumulation of NO_3_^−^–N in the study area, as well as effectively simulate the accumulation of NH_4_^+^–N. This may be due to deficiencies in the APSIM model structure, inadequate characterization of mineralization and nitrification processes, and insufficient consideration of soil adsorption effects on NH_4_^+^–N, leading to a systematic underestimation of NH_4_^+^–N in soil layers.

### 3.2. Effects of Precipitation Pattern and Nitrogen Application Rate on Soil Nitrogen Residuals

Soil NO_3_^−^–N and NH_4_^+^–N, as the primary forms of nitrogen absorbed and utilized by crops, serve as key indicators for assessing the nitrogen supply status in the soil. Different nitrogen application rates have significant effects on soil NO_3_^−^–N and NH_4_^+^–N [39]. This study found that the residual levels of soil NO_3_^−^–N and NH_4_^+^–N in alfalfa farmland increased with higher nitrogen fertilizer application. Excessive nitrogen fertilizer application did not significantly increase alfalfa yield; rather, it led to a substantial accumulation of NO_3_^−^–N and NH_4_^+^–N in the soil. Notably, the residual levels at the 240 kg/ha nitrogen application rate were generally higher than those at other rates. This result is consistent with the research by Zhao et al. [40], both indicating that exogenous nitrogen addition has a significant promoting effect on soil NO_3_^−^–N and NH_4_^+^–N content. Similarly to the results of Jin et al. [1] on NO_3_^−^–N residuals in corn farmland soil, but with notably different content rates, this difference may be attributed to the fact that alfalfa is a perennial legume plant that forms symbiotic relationships with rhizobia, enabling it to convert atmospheric nitrogen into NH_4_^+^–N for its own use. Therefore, it has lower dependence on soil nitrogen pools and typically requires minimal nitrogen fertilizer input. In contrast, corn is a high-yielding annual cereal crop with substantial and concentrated nitrogen demands throughout the growing season, especially during the jointing-to-heading stage. Nitrogen fertilizer not absorbed by the crop remains in the soil as NO_3_^−^–N and NH_4_^+^–N, causing residual accumulation after “luxury uptake” [41]. Additionally, the soil in corn fields often remains bare or sparsely covered for extended periods before planting and after harvest. Under these conditions, NO_3_^−^–N in the soil is highly susceptible to leaching downward with water movement. Yang et al. [42] conducted pot and field experiments on tobacco plants, demonstrating that at nitrogen application rates of 105 kg/ha and 126 kg/ha, soil NO_3_^−^–N content changed significantly, while NH_4_^+^–N content showed no statistically significant differences. This may be attributed to Yang’s addition of biochar alongside nitrogen application, which increased the soil carbon–nitrogen ratio. This increase stimulated microbial growth, leading to preferential absorption and retention of NH_4_^+^–N for microbial metabolism. As a result, the free NH_4_^+^–N content in the soil decreased, rendering its changes statistically insignificant [42,43].

The residual amounts of soil NO_3_^−^–N and NH_4_^+^–N are also affected by precipitation patterns. Zhai et al. [44] conducted a scientific nitrogen fertilization experiment for maize under various precipitation patterns in drip irrigation conditions in the Ningxia region. Their findings demonstrated that peaks in soil NO_3_^−^–N content are closely associated with the amounts of precipitation. Compared with dry years, the peak residual NO_3_^−^–N levels in wet years appeared in deeper soil layers. In this study, the residual amounts of soil NO_3_^−^–N and NH_4_^+^–N were found to be in the order of wet year < normal year < dry year. The trends of NO_3_^−^–N and NH_4_^+^–N residues showed a steeper increase in dry years, while normal years and wet years showed more gradual change trends. This study also found that alfalfa in dry year types exhibited the highest residual levels of soil NO_3_^−^–N and NH_4_^+^–N, which aligns with the observations made by Guo et al. [45]. This may be because, under dry years, the inhibitory effects of water stress on alfalfa root growth and nutrient absorption capacity, combined with limitations in soil nitrogen transport rates through mass flow and diffusion, jointly affect the soil nitrogen cycling process. Simultaneously, the activity of microbial-driven nitrogen turnover decreases, further weakening the intensity of nitrification-denitrification in soil and reducing alfalfa’s nitrogen absorption efficiency. The combined effects of these factors lead to an increase in the residual amounts of nitrate nitrogen and ammonium nitrogen in soil to peak levels.

### 3.3. Effects of Precipitation Pattern and Nitrogen Application Rate on Alfalfa Yield and Nitrogen Fertilizer Use Efficiency

Nitrogen and water jointly regulate key physiological processes, including nutrient absorption, translocation, and osmotic adjustment. Consequently, two essential limiting factors are indispensable for crop physiological metabolism and yield formation [46]. According to the simulation results of the APSIM–Lucerne model, alfalfa yield exhibits a non–linear response to nitrogen fertilizer application under varying precipitation conditions. Specifically, as nitrogen application rates increase, the yield initially rises before subsequently declining. Similar trends were also found in studies by Chang et al. [8] on alfalfa and Ma et al. [47] on cotton. Appropriate nitrogen applications can effectively meet the nitrogen demands during critical growth periods of alfalfa, enhancing its drought resistance and promoting photosynthesis and biomass accumulation [48]. Insufficient nitrogen application results in nitrogen nutrition limitations, leading to reduced chlorophyll content and decreased photosynthetic rates, which significantly affect alfalfa yield and quality. Excessive nitrogen input inhibits the activity of plant nitrate reductase and sucrose reductase, disrupts nitrogen metabolism, and causes soil salinization and nutrient imbalance, ultimately slowing crop growth [49]. Furthermore, this study also found that under conditions of equal nitrogen input, alfalfa dry matter yield showed significant differentiation along the precipitation year–type gradient, with wet years significantly higher than normal years, and dry years the lowest. The suitable nitrogen application range for wet years was 150–183 kg/ha, for normal years 135–160 kg/ha, while the nitrogen critical threshold for dry years decreased to 107–140 kg/ha, indicating that water availability significantly regulates the upper limit of nitrogen demand for alfalfa. Similarly, studies by Kothari et al. [50] on saffron (*Crocus sativus* L.) in the western Himalayas and He et al. [51] on rapeseed in the middle Yangtze River ecological experimental area also showed that precipitation resources are important for crop growth, with higher precipitation leading to higher crop yields. Unlike the study by Ru et al. [52] based on the APSIM model on nitrogen application thresholds for wheat (dry years 182.73 kg/ha, normal years 208.26 kg/ha, and wet years 211.15 kg/ha), the difference may be due to significant differences in soil water conditions in the regions where the two studies were conducted under different precipitation patterns. Appropriate soil water can balance the water–to–air ratio in the soil, ensuring that crop roots can effectively absorb nutrients and water, ultimately improving yield and quality. Excessive or insufficient water leads to inadequate soil oxygen, impairing root respiration and nutrient absorption. This imbalance inhibits microbial decomposition of organic matter and slows nutrient release. Prolonged waterlogging can lead to root rot, yellowing of the crops, and, in severe cases, even death [53,54]. Alfalfa, leveraging its unique nitrogen–fixing ability through root nodules as a legume, can self–supplement nitrogen nutrition, thereby reducing dependence on nitrogen fertilizer [29]. In contrast, wheat, as a grass family crop, relies entirely on soil for its nitrogen supply, requiring greater external nitrogen fertilizer input during growth. Therefore, its demand for external nitrogen fertilizer is higher than that of alfalfa.

Rational nitrogen fertilizer management strategies are crucial for regulating nitrogen accumulation in crops. The precipitation pattern, nitrogen application rate, and their interactive effects collectively influence crop nitrogen absorption and utilization. This study shows that the PFPN continuously decreases with increasing nitrogen application rates, while the ANUE exhibits a parabolic response pattern with an initial increase followed by a decrease, which is consistent with the research results of Lu et al. [55]. This may be because the initial increase in nitrogen application effectively alleviates nutrient limitations, significantly enhancing yield and improving ANUE. However, continued application can lead to diminishing returns, nutrient imbalances (such as disturbances in carbon-nitrogen metabolism), inhibition of self–nitrogen fixation, and potential issues like lodging and pest diseases, which result in a slowdown or even a decline in yield growth. Consequently, ANUE decreases, while PFPN continues to dilute and decline. Besides the nitrogen application rate, alfalfa’s nitrogen fertilizer use efficiency is also closely related to the precipitation pattern. This study shows that at the same nitrogen application level, the PFPN in alfalfa was highest in wet years, intermediate in normal years, and lowest in dry years (Figure 5). Meanwhile, when the nitrogen application rate was within the optimal range corresponding to each year type, the ANUE of alfalfa also showed a consistent pattern: wet years > normal years > dry years (Figure 5). This may be because an adequate water supply improves soil aeration, promotes alfalfa root development, and enhances the plant’s efficiency in nitrogen absorption and utilization. Under insufficient water supply or water stress conditions, the migration of nitrogen toward the alfalfa rhizosphere in the soil is hindered, inhibiting alfalfa rhizosphere growth and its physiological activity, thus significantly reducing the crop’s nitrogen absorption and utilization efficiency. Therefore, compared with wet years, alfalfa’s PFPN and ANUE in dry years are significantly reduced, and the optimal nitrogen application level correspondingly decreases.

Due to the limitations of field experimental data, this study focused solely on one variety of alfalfa, “Longdong” purple alfalfa, and calibrated the APSIM–Lucerne model using a manual trial-and-error approach. To further enhance the model’s simulation accuracy and application potential in the study region, subsequent research should: (1) expand the model’s capability to simulate dynamic responses and precisely model different varieties and key agronomic practices, such as water and fertilizer management; and (2) replace the traditional trial-and-error method with intelligent optimization algorithms, such as the mixed frog-leaping algorithm and particle swarm optimization, to improve the objectivity and precision of parameter calibration.

## 4. Materials and Methods

### 4.1. Description of the Study Area

**Figure 8:**
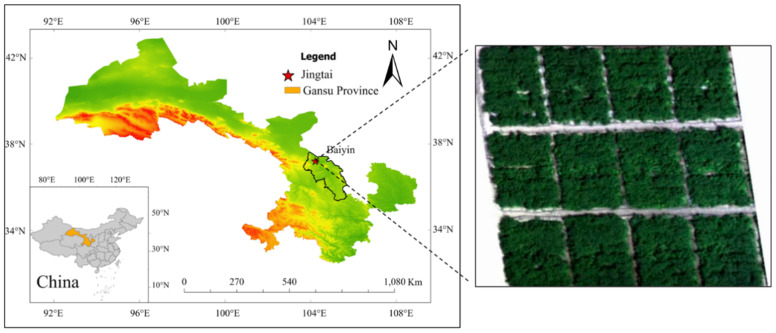
Geographic location of the study area.

**Figure 9:**
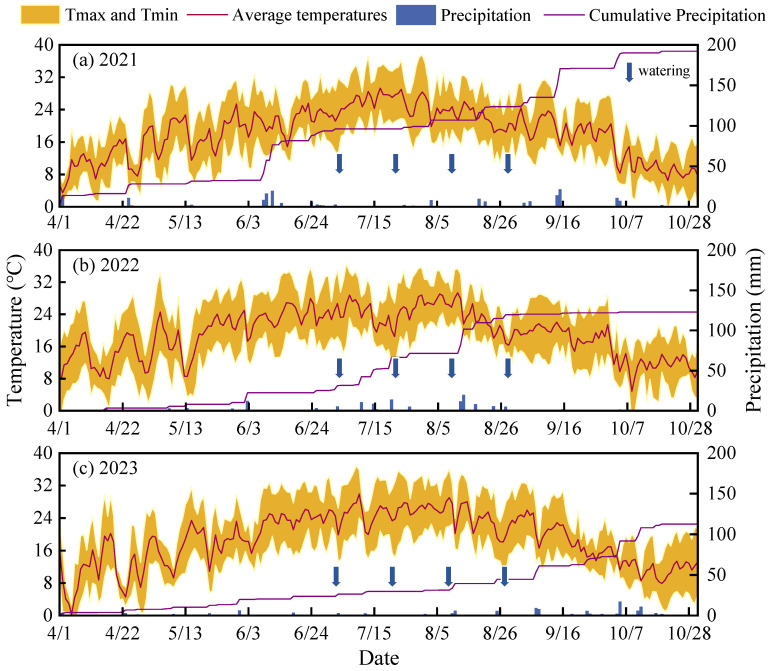
The distribution characteristics of daily precipitation and temperature during the experimental period.

This study was conducted from April to October 2021–2023 at the Jingtaichuan Electric Power Lift Irrigation Water Resources Utilization Center Irrigation Experimental Station in Gansu Province (37°23′ N, 104°08′ E, elevation 2028 m, Figure 8). The region’s annual precipitation, evaporation, sunshine duration, solar radiation, annual average temperature, and frost-free period are 185 mm, 3028 mm, 2652 h, 6.18 × 10^5^ J/cm^2^, 8.5 °C, and 191 days, respectively. The test soil was sandy loam, with a soil bulk density of 1.61 g/cm^3^, field water-holding capacity of 24.1%, and pH value of 8.11. The soil organic matter, total nitrogen, total phosphorus, total potassium, alkali-hydrolyzable nitrogen, available phosphorus, and available potassium contents were 6.09 g/kg, 1.62 g/kg, 1.32 g/kg, 34.03 g/kg, 55.2 mg/kg, 26.31 mg/kg, and 173 mg/kg, respectively. During the three-year study period, all meteorological data were continuously monitored and recorded automatically by a micro-scale smart agriculture weather station installed at the experimental site. The observed values for annual precipitation and daily average temperature for each year were: 192.22 mm and 19.09 °C (Year 1), 122.91 mm and 18.78 °C (Year 2), and 112.42 mm and 19.11 °C (Year 3), respectively (Figure 9).

### 4.2. Experimental Design

The test material used in this study was Longdong purple flower alfalfa (referred to as alfalfa). The experiment comprised four nitrogen application rates (pure nitrogen, urea, with a nitrogen mass fraction of 46.4%), including N0 (0 kg/ha), N80 (80 kg/ha), N160 (160 kg/ha), and N240 (240 kg/ha). Each treatment was replicated three times, for a total of 12 plots, with a plot area of 42.9 m^2^ (5.5 m × 7.8 m). Alfalfa was established in April 2021. The field was leveled 10 days prior to sowing. Row-seeding was employed with a row spacing of 30 cm, a sowing depth of 30 mm, and a seeding rate of 22.5 kg/ha. In 2021, nitrogen fertilizer was applied at a 6:4 ratio before alfalfa seeding and after the first cutting, respectively. In 2022–2023, it was applied at a 6:2:2 ratio during the regreening stage, after the first cutting, and after the second cutting, respectively. Phosphate fertilizer (diammonium phosphate, with a P_2_O_5_ mass fraction of 16%) and potassium fertilizer (potassium chloride, with a K_2_O mass fraction of 50%) were both applied as basal fertilizer in a single application at the time of seeding each year (or during the green-up period), each at a rate of 50 kg/ha. Water management throughout the entire growing period was conducted using a drip irrigation system. The water supply pipeline was equipped with control valves and a water meter with an accuracy of 0.001 m^3^ to precisely control the irrigation amount for each application. Alfalfa maintained full irrigation status throughout the entire growing season, and all irrigation and other field management measures followed the local standardized alfalfa cultivation procedures.

### 4.3. Measurement Indices and Methods

#### 4.3.1. Soil NO_3_^−^–N and NH_4_^+^–N Content (mg·kg^−1^)

During the research period of 2021–2023, the NO_3_^−^–N and NH_4_^+^–N contents in the 0–120 cm soil layer were measured once after the last cutting of alfalfa each year. Soil samples were collected using a stratified sampling method, with each 20 cm layer sampled from top to bottom. Soil NO_3_^−^–N content was determined using an ultraviolet–visible spectrophotometer (Beijing Puxi General Instrument Co., Ltd., T6 New Century, Beijing, China), while soil NH_4_^+^–N content was determined using the indophenol blue colorimetric method [56].

#### 4.3.2. Soil NO_3_^−^–N and NH_4_^+^–N Residual Content (kg/ha)

The calculation formula for residual soil nitrate and ammonium nitrogen is as follows [57]:

(1) $$\mathrm{NR}=\gamma_{i}h_{i}N_{i}/10$$

In the formula, *h_i_* represents the thickness of the *i*-th soil layer (cm), $\gamma_{i}\;$ represents the soil bulk density of the *i*-th soil layer (g/cm^3^), and *N_i_* represents the soil NO_3_^−^–N (NH_4_^+^–N) content of the *i*-th soil layer (mg/kg).

#### 4.3.3. Yield (Y, t·ha^−1^)

To coordinate alfalfa hay yield, nutritional quality, and post-harvest regeneration performance, this experiment uniformly performed cutting at the early flowering stage of alfalfa and controlled stubble height at 5 cm. During each cutting, representative 1 m × 1 m (1 m^2^) sample plots were selected within each experimental plot. The harvested fresh grass samples were dried as follows: after blanching at 105 °C for 30 min, they were dried at 75 °C to constant weight. After the samples cooled, their dry weight was measured, and the hay yield was calculated.

#### 4.3.4. Nitrogen Fertilizer Use Efficiency (NUE)

In agricultural management, nitrogen fertilizer use efficiency is often quantified through indicators such as nitrogen fertilizer partial factor productivity and nitrogen fertilizer agronomic efficiency.

Partial factor productivity of nitrogen (*PFPN*, kg/kg):

(2) PFPN=Y/F

In the formula, *Y* represents hay yield (t/ha), and *F* represents nitrogen fertilizer application rate (kg/ha).

Agronomic nitrogen use efficiency (*ANUE*, kg/kg):

(3) $$\mathrm{ANUE}=\;(Y_{\mathrm{NPK}}-Y_{\mathrm{PK}})/F$$

In the formula, *Y_NCK_* represents hay yield under nitrogen treatment (t·ha^−1^), *Y_CK_* represents hay yield under the no-nitrogen control treatment, and *F* represents nitrogen fertilizer application rate (kg/ha).

### 4.4. Classification of Different Precipitation Patterns

Using the daily precipitation data from the study area, the precipitation during the alfalfa growth period and the drought index (*D*) were calculated. Based on the D values, the precipitation data for 25 years (2000–2024) in the study area were classified into three types: dry years, normal years, and wet years. The specific calculation formula is as follows [58]:

(4) D= (C−A)/σ

In the formula, *D* is the dry index (*D* < −0.35 indicates dry years, *D* > 0.35 indicates wet years, and −0.35 ≤ *D* ≤ 0.35 indicates normal years); *C* is the precipitation during the growth period (mm); *A* is the average precipitation during the growth period (mm); and σ is the standard deviation. The specific classification of year types is shown in Table 1.

### 4.5. Scenario Design

The scenario design for the APSIM model is consistent with the field experiment setup, with additional fertilization gradients of N120 (120 kg/ha), N140 (140 kg/ha), N180 (180 kg/ha), and N200 (200 kg/ha). The simulation period is from 1 January 2000 to 31 December 2024.

### 4.6. Model Construction and Applicability Verification

#### 4.6.1. Construction of the APSIM–Lucerne Model

**Figure 10:**
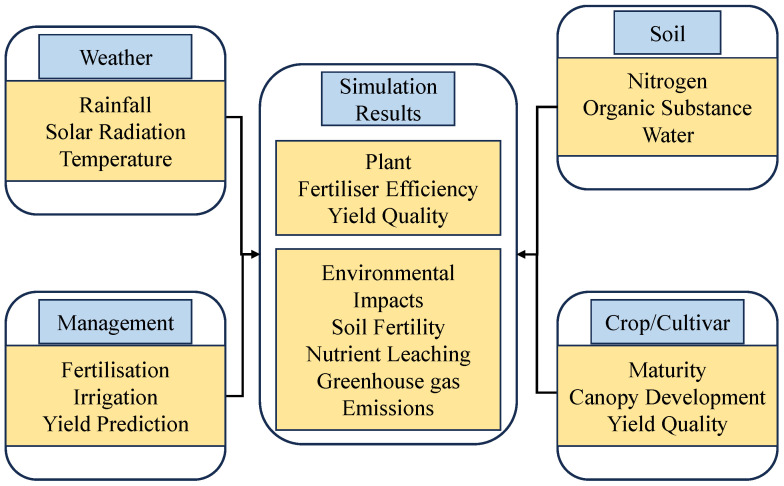
Model structure diagram. In the figure, blue represents different modules of the model, while yellow indicates specific metrics for each module.

The APSIM–Lucerne model primarily includes four main input data categories: meteorological conditions, soil characteristics, crop parameters, and field management. It effectively simulates the changes in crop growth, yield formation, and soil nutrient indicators under different scenario combinations [59]. The operating framework of the APSIM model is shown in Figure 10. The meteorological data used in this study came partly from a small weather station at the experimental site and partly from the Gansu Provincial Meteorological Bureau, and includes daily maximum temperature (°C), daily minimum temperature (°C), daily rainfall (mm), sunshine duration (h), daily solar radiation (MJ/m^2^), average annual temperature (Tav), and monthly temperature amplitude (Amp), calculated using TAV_AMP program [60]. The soil data (Table 2) mainly includes indicators such as layered soil bulk density (BD), saturated water content (SAT), field capacity (DUL), wilting point (LL15), and air-dry soil water content (Air dry), among others.

Parameter calibration of the APSIM–Lucerne model was based on field trial data collected from 2021 to 2023 and was guided by the study of Li et al. [61] (Table 3). The optimal parameters were identified through iterative trial-and-error testing and refinement.

#### 4.6.2. Validation Method

To assess the adaptability of the APSIM model in the study area, this study selected the coefficient of determination (*R*^2^), root mean square error (RMSE), normalized root mean square error (NRMSE), index of agreement (D), and mean absolute error (MAE) for validation [62,63]. The relevant calculation formulas are as follows:

(5) $$R^{2}=1-\frac{\sum_{i=1}^{n}{\left(Y_{sim,i}-Y_{obs,i}\right)}^{2}}{\sum_{i=1}^{n}{\left(Y_{obs,i}-{\bar{Y}}_{obs,i}\right)}^{2}}$$

(6) $$\mathrm{RMSE}=\sqrt{\frac{\sum_{i=1}^{n}{\left(Y_{sim,i}-Y_{obs,i}\right)}^{2}}{n}}$$

(7) $$\mathrm{NRMSE}(\%)=\frac{\mathrm{RMSE}}{{\bar{Y}}_{obs}}\times 100\%$$

(8) $$D=1-\frac{\sum_{i=1}^{n}{\left(Y_{sim,i}-Y_{obs,i}\right)}^{2}}{\sum_{i=1}^{n}{\left(\left|Y_{sim,i}-\left.{\bar{Y}}_{obs}\right|+\left|Y_{obs,i}-\left.{\bar{Y}}_{obs}\right|\right.\right.\right)}^{2}}$$

(9) $$MAE=\frac{\sum_{i=1}^{n}\left|Y_{sim,i}–Y_{obs,i}\right|}{n}$$

In the formulas, *Y_sim_* represents the simulated values, *Y_obs_* represents the measured values, and, represents the average of the measured values, and n represents the sample size.

### 4.7. Data Processing

Data processing and graphical visualization were performed using Excel 2016 and Origin 2023. Statistical analysis of all data was conducted using one-way analysis of variance (ANOVA) in SPSS 2.1.0, with a significance threshold of *p* = 0.05.

## 5. Conclusions

(1) The *R*^2^ and NRMSE for yield verification of the APSIM–Lucerne model were 0.91 and 6.55%, respectively, while the *R*^2^ and NRMSE for NO_3_^−^–N and NH_4_^+^–N residue verification were 0.67, 0.86, 21.76%, and 24.03%, respectively.
(2) Under different precipitation patterns, alfalfa yield and ANUE exhibited a non-linear response, initially increasing and then decreasing with higher nitrogen application rates. As nitrogen application levels increased, significant accumulation of soil residual NO_3_^−^–N and NH_4_^+^–N was observed; however, the PFPN consistently declined with increasing nitrogen application rates.
(3) Based on simulations using the calibrated APSIM–Lucerne model, the optimal nitrogen application thresholds for alfalfa under dry, normal, and wet years are in the ranges of 107–140, 135–160, and 150–183 kg/ha, respectively.

In summary, the APSIM–Lucerne model in this study can effectively simulate alfalfa yield, NO_3_^−^–N, and NH_4_^+^–N residue under different precipitation patterns in the Yellow River irrigation area of Gansu Province. In future research, the APSIM–Lucerne model can be combined with vegetation indices and leaf area index data retrieved from remote sensing to achieve real–time calibration of alfalfa growth status and yield prediction. An integrated precision fertilization intelligent management system for alfalfa that fuses satellite remote sensing, unmanned aerial vehicle (UAV) close–range sensing, and ground-based measurement data—a ‘satellite–UAV–ground’ (star–machine–ground) integrated system—can be established.

## Figures and Tables

**Table 1:** Different precipitation patterns.

Model Year	Average Precipitation in Growth Period (mm)	Years	Year
Dry year	135.10	13	2000, 2004, 2005, 2006, 2008, 2009, 2010, 2012, 2013, 2015, 2020, 2022, 2023
Normal year	212.25	2	2001, 2021
Wet year	271.31	10	2002, 2003, 2007, 2011, 2014, 2016, 2017, 2018, 2019, 2024

**Table 2:** Main soil parameters.

Parameters	Soil Layer (cm)					
	0–20	20–40	40–60	60–80	80–100	100–120
BD (g/cm^3^)	1.270	1.350	1.280	1.140	1.240	1.300
SAT (mm/mm)	0.460	0.431	0.467	0.520	0.482	0.459
DUL (mm/mm)	0.197	0.213	0.241	0.278	0.233	0.253
LL15 (mm/mm)	0.061	0.069	0.075	0.086	0.072	0.078
Air dry (mm/mm)	0.010	0.030	0.070	0.070	0.070	0.070
Soil pH	8.095	8.120	8.410	8.540	8.700	8.700
Swcon (0–1)	0.600	0.600	0.500	0.500	0.500	0.500
LucerneLL (mm/mm)	0.290	0.290	0.300	0.310	0.320	0.330
LucerneKL (d^−1^)	0.100	0.100	0.090	0.090	0.090	0.090
LucerneXF (0–1)	1.000	1.000	1.000	1.000	1.000	1.000

**Table 3:** Key parameters of the APSIM–Lucerne model.

Parameters	Value	Unit
tt_emerg_to_endjuv	550	°C·d
tt_endjuv_to_init	610	°C·d
tt_endjuv_to_init	260	°C·d
Photoperiod required for floral initiation	>10	h
Radiation use efficiency	1.8	g/MJ
Stem weight	0~5	g/plant
Plant height	0~5000	mm
Summer_U	6	mm
Summer Cona	3.5	mm

tt_emerg_to_endjuv: thermal time from emergence to end of juvenile; tt_endjuv_to_init: thermal time from end juvenile to floral initiation; tt_endjuv_to_init: thermal time from initiation to full-blooming; Summer_U: cropping area.

## Data Availability

All data supporting this study are included in the article.
